# Correlation between hearing loss and mild cognitive impairment in the elderly population: Mendelian randomization and cross-sectional study

**DOI:** 10.3389/fnagi.2024.1380145

**Published:** 2024-06-07

**Authors:** Tong Xu, Tao Zong, Jing Liu, Le Zhang, Hai Ge, Rong Yang, Zongtao Liu

**Affiliations:** ^1^Affiliated Qingdao Third People’s Hospital, Qingdao University, Department of Otorhinolaryngology Head and Neck, Qingdao, China; ^2^Affiliated Qingdao Third People’s Hospital, Qingdao University, Department of Clinical Laboratory, Qingdao, China

**Keywords:** Mendelian randomization, mild cognitive impairment, hearing loss, tinnitus, risk

## Abstract

**Background:**

Hearing loss and tinnitus have been linked to mild cognitive impairment (MCI); however, the evidence is constrained by ethical and temporal constraints, and few prospective studies have definitively established causation. This study aims to utilize Mendelian randomization (MR) and cross-sectional studies to validate and analyze this association.

**Methods:**

This study employs a two-step approach. Initially, the genetic data of the European population from the Genome-wide association studies (GWAS) database is utilized to establish the causal relationship between hearing loss and cognitive impairment through Mendelian randomization using the inverse variance weighted (IVW) method. This is achieved by identifying strongly correlated single nucleotide polymorphisms (SNPs), eliminating linkage disequilibrium, and excluding weak instrumental variables. In the second step, 363 elderly individuals from 10 communities in Qingdao, China are assessed and examined using methods questionnaire survey and pure tone audiology (PTA). Logistic regression and multiple linear regression were used to analyze the risk factors of MCI in the elderly and to calculate the cutoff values.

**Results:**

Mendelian randomization studies have shown that hearing loss is a risk factor for MCI in European populations, with a risk ratio of hearing loss to MCI loss of 1. 23. The findings of this cross-sectional study indicate that age, tinnitus, and hearing loss emerged as significant risk factors for MCI in univariate logistic regression analysis. Furthermore, multivariate logistic regression analysis identified hearing loss and tinnitus as potential risk factors for MCI. Consistent results were observed in multiple linear regression analysis, revealing that hearing loss and age significantly influenced the development of MCI. Additionally, a notable finding was that the likelihood of MCI occurrence increased by 9% when the hearing threshold exceeded 20 decibels.

**Conclusion:**

This study provides evidence from genomic and epidemiological investigations indicating that hearing loss may serve as a risk factor for cognitive impairment. While our epidemiological study has found both hearing loss and tinnitus as potential risk factors for cognitive decline, additional research is required to establish a causal relationship, particularly given that tinnitus can manifest as a symptom of various underlying medical conditions.

## Introduction

Mild cognitive impairment (MCI) serves as a transitional phase between typical aging and the onset of clinical dementia, characterized by a mild decline in cognitive abilities, particularly in memory (Cox and Wallace, 2022; Zhao et al., 2023). Individuals with MCI face a heightened risk of developing Alzheimer’s disease (AD), with approximately 32% progressing to dementia within a median timeframe of 2 years (Jafari et al., 2019; Jia et al., 2019; Clark and Swanepoel, 2021; Babulal et al., 2022). Consequently, the identification, assessment, and intervention of MCI warrant further investigation in research endeavors to effectively delay and avert the onset of AD.

Over 1.5 billion individuals globally, constituting approximately 20% of the world’s population, experience varying degrees of hearing loss, with 430 million individuals exhibiting moderate to severe hearing loss (Cunningham and Tucci, 2017; D'Haese et al., 2023). The incidence of hearing loss escalates significantly with advancing age, rising from 12.7% at 60 years to 58.6% at 90 years. Moreover, a substantial portion (58%) of individuals with disabling hearing loss are aged 60 years and above (Lin et al., 2013; Cunningham and Tucci, 2017; Clark and Swanepoel, 2021; McMahon et al., 2021; Zhang et al., 2022; D'Haese et al., 2023).

Hearing loss (HL) is a prevalent disability in today’s increasingly aging society and poses a serious threat to the physical and mental health of older people (Bowl and Dawson, 2019). Hearing loss is the most common chronic sensory impairment in older people, with disabling hearing loss occurring in approximately 50% of people over the age of 70 years, and it is one of the three most common health problems in older people (Ronnberg et al., 2013). The World Health Organization predicts that the global population aged over 65 years will reach 1 billion by 2050 and that hearing loss will become a major public health problem (Lin et al., 2013). In addition to its widespread occurrence, hearing impairment can have a multifaceted effect on elderly individuals and is consequently receiving increasing recognition. Firstly, hearing loss may result in challenges with speech and communication, thereby diminishing older individuals’ verbal communication capabilities (Rutherford et al., 2018). Secondly, hearing loss can precipitate feelings of social isolation, despondency, solitude, and various psychological issues, including depression (Chern and Golub, 2019). Importantly, hearing loss can also lead to cognitive impairment (Livingston et al., 2017). According to Lancet Dementia Commission 2017 and 2020, hearing loss is the greatest potential risk factor for dementia (Livingston et al., 2020), and it is projected that if hearing loss was eliminated, the prevalence of dementia would be reduced by 8%. If the prevalence of each risk factor for hearing loss was reduced by 10 to 20% every 10 years, the number of dementia diagnoses worldwide could be reduced by 8.8 to 16.2 million by 2050 (Czornik et al., 2022).

Tinnitus is a common symptom of the auditory system, which is mainly caused by other diseases, such as noise, hearing loss and mental stress. In Europe and the United States, 10 to 15 percent of people are affected by tinnitus for some time, and the prevalence increases with age (Mazurek et al., 2022). Recent evidence suggests a link between tinnitus and impairment in all aspects of cognitive function. Both hearing loss and tinnitus can affect mental health and contribute to depression, stress, and depression (Jarach et al., 2022; Lau et al., 2022). This study included tinnitus in the community survey project, providing new evidence for the study of tinnitus and cognitive impairment.

Previous observational studies and meta-analyses have identified hearing loss as a possible risk factor for cognitive impairment and dementia, but confounding could not be excluded (Loughrey et al., 2018; Jafari et al., 2019). Recent studies have found that hearing loss is associated with cognitive decline, brain atrophy and tau protein by genetic association analysis using data from different databases, which again confirms that hearing loss is a risk factor for cognitive decline and dementia (Wang et al., 2022). However, there is no direct evidence that hearing loss is causally related to cognitive impairment and dementia. These observational and bioinformatic findings need to be confirmed in randomized, controlled trials and animal studies that are time - and ethically demanding. A 2022 randomized controlled trial at four community study sites in the United States of America in adults aged 70 to 84 years with untreated hearing loss and no severe cognitive impairment. The findings suggest that hearing interventions may reduce cognitive change over 3 years in an older population at increased risk of cognitive decline, but not in a population at reduced risk of cognitive decline (Lin et al., 2023). Therefore, more evidence is needed to confirm the direct relationship between hearing loss and cognitive impairment. Mendelian randomization (MR) uses genetic tools to provide new evidence for confirming the causal relationship between exposure factors and diseases (Emdin et al., 2017; Ji et al., 2024; Levin and Burgess, 2024). Mendelian randomization is widely used in epidemiological etiology research because it can avoid the ethical and time limitations of randomized controlled trials and the interference of confounding factors in case–control trials. Based on large samples of genetic and phenotypic data, MR Screens single nucleotide polymorphisms (SNPS) that are strongly associated with exposure factors but not related to outcomes and confounding factors, and uses them as instrumental variables to assess the causal relationship between exposure factors and outcomes (Larsson et al., 2023).

**Figure 1:**
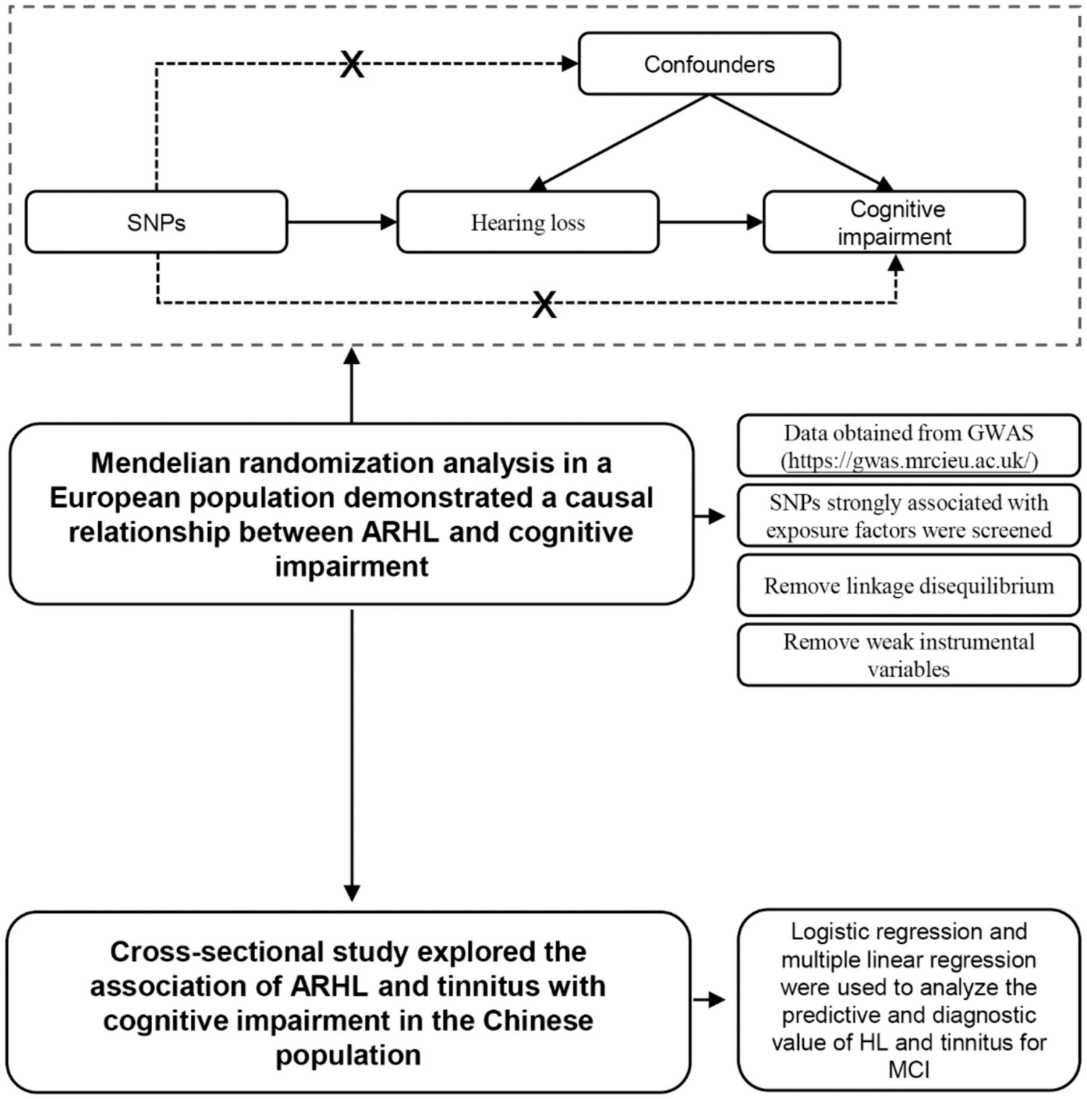
Flow chart of Mendelian randomization and cross-sectional studies and the principle of Mendelian randomization.

This study posited that hearing loss, tinnitus, weight, and other variables may serve as potential risk factors for cognitive impairment. Genetic and epidemiological methodologies were employed to investigate these risk factors in European and Chinese populations, with logistic regression and linear regression utilized to analyze the relationship between these factors (Figure 1). The findings of this study aim to contribute new evidence to the understanding of the link between hearing loss, tinnitus, and cognitive impairment.

## Methods

### Mendelian randomization analysis between hearing loss and cognitive function

Data of hearing loss (ebi-a-GCST90018857, including 489,493 European adult subjects and consisted of 14,654 cases and 474,839 controls) which obtained from GWAS^1^ (S1) is defined as exposure data, which was subjected to selection of SNPs for strongly correlated instrumental variables (*p* < 2 × 10^−7^), followed by analyses to remove linkage disequilibrium (SNPs within 10,000 kb of the highest SNPs with significance at *r*^2^ < 0.001) were removed and filtering of weak instrumental variables (*F*-value >10) (S2) (Storey and Tibshirani, 2003). Data of cognitive functions (ieu-b-4837, including 9,997 European adult subjects) were defined as outcome data, which was subjected to extraction of instrumental variable SNPs and then merged with exposure data and analyzed by Mendelian randomization.

Two-sample MR Analysis was used in this study. Five methods were used for Mendelian analysis, namely MR Egger method, Weighted median method, Inverse variance weighted method, Simple mode method, and Weighted mode method. Inverse-variance weighting was used as the primary analysis method. Inverse variance weighting methods estimate the causal effects of genes on traits by weighting the causal effects of different genetic variants on traits and then combining the estimated effects after weighting. The advantage of inverse variance weighting method is to reduce the influence of sample size, improve the estimation accuracy and reduce bias, so it is used as the main analysis method (Sanderson, 2021). MR-egger method and weighted median method were used as supplements, and the Beta value was calculated to judge whether there was consistency in the results of MR Analysis to enhance the robustness of causality. MR-Egger method can calculate direct and indirect effects, evaluate the multiple effects of genetic variation on the results, adjust the confounding bias to a certain extent, and improve the accuracy of causality estimation (Burgess and Thompson, 2017). The weighted median method mainly assigns different weights to different genetic variants, thereby reducing the impact of extreme genetic variation on causal inference and improving the stability of the results. In addition, the weighted median method also has the advantages of increasing the estimation accuracy, wide applicability and strong flexibility (Hartley et al., 2022). The results of Mendelian randomization were finally tested for heterogeneity (S3), sensitivity and pleiotropy (S4), and funnel plots, forest plots and scatter plots were created.

### Population of the cross-sectional study

All of the subjects were recruited from residents of Liking District, Qingdao, aged 60 years or older. Exclusion criteria were as follows: having dementia, bipolar disorder, schizophrenia, or other developmental or neurodegenerative disorders or that prevented performing cognitive assessment tests, and currently wearing hearing aids such as hearing aids or cochlear implants. Recruitment and testing took place from May to July 2022. The study was approved by the Ethics Committee of the Third People’s Hospital of Qingdao, affiliated with Qingdao University. All the procedures were carried out in accordance with the approval, and the participants provided written informed consent.

Questionnaire survey and pure tone audiometry were conducted among 363 elderly people in 10 communities in Qingdao through recruitment and promotion in community health centers. Gender, age, height, body mass index (BMI), hypertension, diabetes, tinnitus, depression, and hearing loss were used as independent variables, and MCI was used as the dependent variable to conduct univariate logistic regression to preliminarily explore the risk factors of MCI. Univariate logistic regression analysis was used to screen out statistically significant independent variables, and then multivariate logistic regression analysis was used to determine the risk factors of MCI. Finally, the cut-off value of Youden index for the diagnosis of MCI was calculated by logistic regression.

### Data collection

Data collection consisted of the following three parts: instrumental measurements, questionnaire, and scale assessment. Height, blood pressure, weight, and hearing test data were obtained from instrumental measurements, depression, and cognitive function data were obtained from scale assessment. Diabetes diagnosis was obtained by asking medical history. The data collection process was completed by clinicians from the Department of Otorhinolaryngology of the Third People’s Hospital of Qingdao affiliated with Qingdao University.

### Scale assessment

All the subjects were assessed on the Hearing Handicap Inventory for the Elderly-Screening. Higher scores on the Hearing Handicap Inventory for the Elderly-Screening indicated greater hearing loss and a scale score of >8 was defined as the presence of hearing loss (Kim et al., 2016). The Montreal Cognitive Assessment Scale (MoCA) has a high sensitivity and specificity for the rapid screening of patients with MCI and is now widely used internationally (Lee et al., 2020). MoCA is divided into eight cognitive domain subscales, of which the immediate memory subscale is not scored and the other seven cognitive domain subscales are scored as follows: 5 points for visuospatial and executive functions, 3 points for language skills, 6 points for attention, 6 points for orientation, 5 points for delayed memory, 3 points for naming, and 2 points for abstraction. The original English version of the MoCA recommended a score of less than 26 for cognitive impairment. In studies on the Chinese population, a score of 26 is generally considered high. There is some variation in the optimal cutoff values used in different diseases and populations. For example, in the case of non-demented vascular cognitive impairment, some studies have shown that the optimal cutoff score for MoCA is 23.5 (Wang et al., 2018). Based on the results of the study on the Chinese population, the MoCA score of 26 was used as the cut-off value in this study (Jia et al., 2021). Chinese elderly have free physical examination in the community every year, and have chronic disease management files, so that they are very aware of their own chronic disease history, so data on diabetes, hypertension, and tinnitus were obtained through patient self-evaluation (Respondents were asked if they heard a non-pulsating cicada, cricket, or whistle for an extended period of time) (Vasilescu and Weisman, 2023). Depression data were obtained by assessment of the Patient Health Questionnaire-9 (PHQ-9) scale, the scale had 9 items, including mood, sleep, appetite, fatigue, self-identity, attention and self-injury. Each item was composed of four options (0 = not at all, 1 = a few days, 2 = more than half of the days, 3 = almost every day). The total score ranged from 0 to 27, and the total score > 4 was considered positive (Levis et al., 2019).

### Hearing test

All the subjects were audiometrically tested by an audiologist for pure tone, with air conduction at 500, 1000, 2000, and 4,000 Hz. The hearing test equipment was Pure audiometer—Clinical diagnostic audiometer (AD226) produced by International Hearing Company, Denmark. If the average of the air conduction thresholds of 500, 1,000, 2000, and 4,000 Hz for the better ear, in accordance with the WHO classification criteria for the degree of hearing loss, was ≤25 decibels hearing level, it was considered normal hearing, while more than 25 decibels hearing level was considered hearing loss.

### Statistical methods

Chi-square test and Mann–Whitney U test were used to compare the differences between groups according to the number of samples and the distribution of data. The effect of the independent variables on cognitive impairment was assessed using the odds ratio (OR) in logistic regression, with an OR > 1 and a statistically significant *p*-value indicating that the variable was a risk factor for cognitive impairment, and an OR < 1 and a statistically significant p-value indicating that the variable was a protective factor for cognitive impairment. Statistical analyses were performed using Stata 12, R 4.2.2 and SPSS (v28.0.1.1) software, and differences were considered statistically significant at *p* < 0.05. The cut-off value was determined based on the Youden index. The Youden index is the sum of sensitivity and specificity minus 1, and the maximum corresponding value is the Youden index, which is the cut-off value.

## Results

### Mendelian randomization

**Figure 2:**
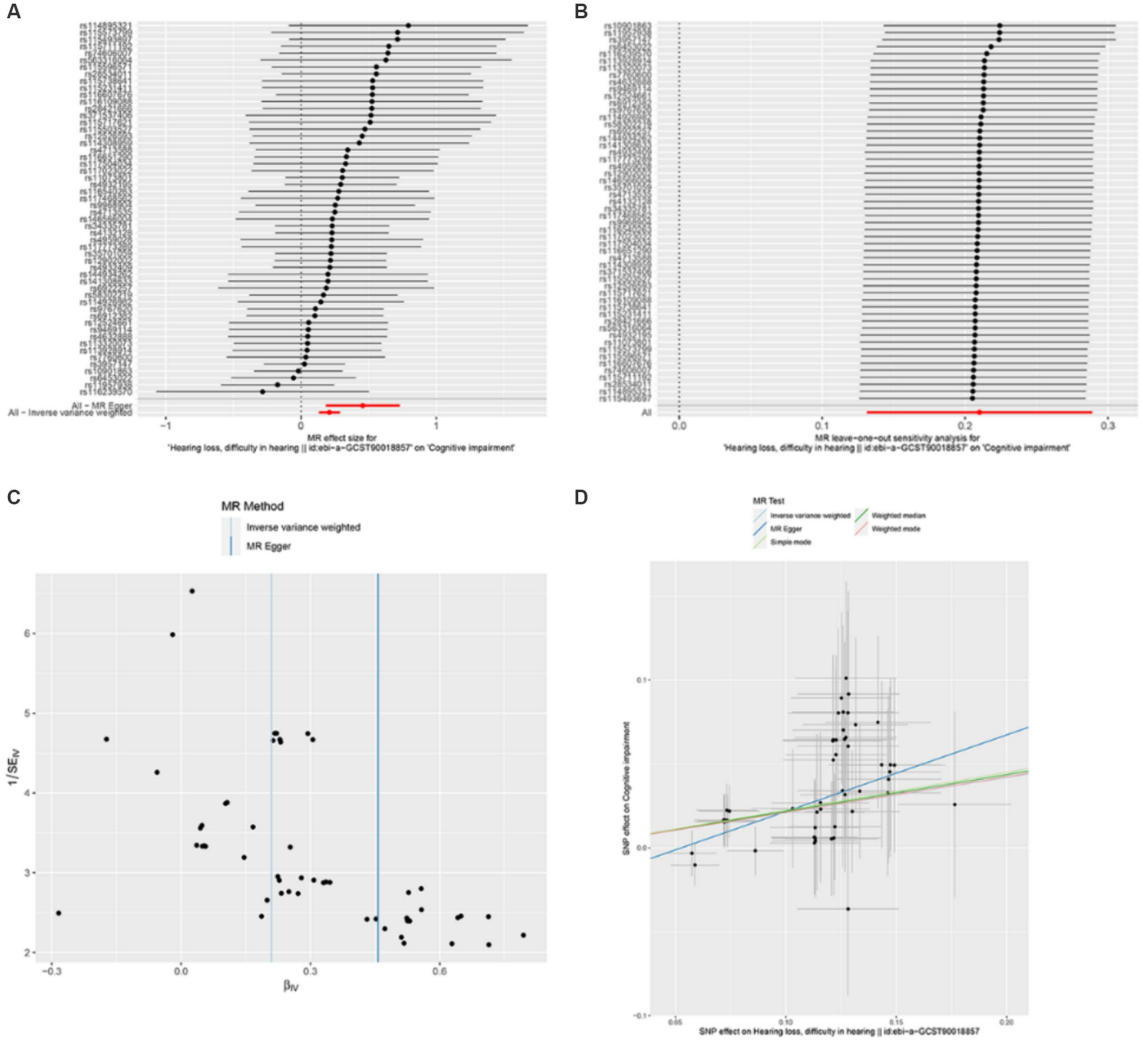
Mendelian randomization analysis between hearing loss and cognitive function. **(A)** is an effect forest plot of Mendelian randomization showing the effect value of Mendelian randomization for each SNP and the total effect value (beta value), which shows that 50 out of 54 effect SNPs have a hazardous effect, 4 SNPs have a protective effect, and the total effect is a hazardous effect. **(B)** is a leave-one-out method to analyze the sensitivity of each SNP. After each SNP was taken out, the results of the remaining SNP analysis remained robust. **(C)** shows the funnel plot to detect bias. The results show that the SNPs are evenly distributed on both sides of the total effect line, demonstrating that the analysis is not significantly biased. **(D)** shows the scatterplot of the five analytical methods of Mendelian randomization. The horizontal coordinate is the effect of SNP on exposure factors and the vertical coordinate is the effect of SNP on the results. All five methods of analysis showed that hearing loss exerts a detrimental effect on cognitive function.

Through Mendelian randomization analysis, we found an instrumental variable consisting of 54 SNPs, thus demonstrating a causal relationship between HL and cognitive impairment. Inverse variance weighted analysis showed an OR of 1.23 with a *p*-value of 1.75 × 10^−7^ (Table 1). The *p*-value for the test of pleiotropy was 0.07 and for the test of heterogeneity was 0.99. Sensitivity analysis and funnel plot of leave-one-out method also showed less bias in Mendelian analysis (Figure 2). Our Mendelian randomization results suggest that HL is a risk factor for cognitive impairment. In order to confirm the results of Mendelian randomization, we conducted a cross-sectional study in a Chinese population for validation.

**Table 1:** Results of Mendelian randomization analysis.

Outcome	Exposure	Method	Number of SNP	*p* value	OR	OR_lci95	OR_uci95
Cognitive impairment	Hearing loss	MR Egger	54	0.0018	1.579	1.201	2.075
Cognitive impairment	Hearing loss	Weighted median	54	9.43E-05	1.243	1.114	1.387
Cognitive impairment	Hearing loss	Inverse variance weighted	54	1.75E-07	1.233	1.140	1.335
Cognitive impairment	Hearing loss	Simple mode	54	0.0600	1.251	0.995	1.573
Cognitive impairment	Hearing loss	Weighted mode	54	0.0279	1.235	1.028	1.484

Mendelian analysis of hearing loss and cognitive impairment was performed using five methods. The results showed that there were four methods with *p* value less than 0.05, and the most important analysis method was Inverse variance weighted (IVW) method with *p* value of 1.75E-07 and OR value of 1.23. The results showed that the risk of cognitive impairment in people with hearing loss was 1.23 times higher than that in the general population.OR, odds ratio; LCI, lower confidence interval; UCI, upper confidence interval.

### Characteristics of the survey population

As shown in Table 2, a total of 363 subjects were included in this study. They were aged 60 to 88 years, with 204 men and 159 women. According to the MoCA scale, 95 of the 363 study subjects had a normal cognitive function and 268 had a cognitive decline. Age, hearing loss, and tinnitus were significantly different between the two groups, while all the other variables were not significantly different.

**Table 2:** Baseline characteristics of the control group and patients with mild cognitive impairment.

		Control group (*n* = 95)	MCI group (*n* = 268)	*p* value
Gender	Male	54	150	0.99^a^
	Female	41	118	
Age at enrollment, mean (Range)		66.4(60–86)	69.8(60–88)	0.02^b^*
Height (Range)		163 (152–186 cm)	164 (150–183 cm)	0.25^b^
Body weight		68.6 (45–93 kg)	66.6 (45–115 kg)	0.5^b^
BMI (Range)		25.4 (17.5–39.7)	24.8 (16.5–42.7)	0.23^b^
Hypertension	Yes	22	88	0.09^a^
	No	73	180	
Diabetes	Yes	16	40	0.74^a^
	No	79	228	
Tinnitus	Yes	19	91	0.01^a^*
	No	76	177	
Depression	Yes	13	48	0.42^a^
	No	82	220	
Hearing loss (Range)		25.4 (10–60)	37.7 (10–89)	<0.01^b^*
MoCA value (Range)		27.2 (26–30)	20.3 (7–25)	<0.01^b^*

The results showed that age, tinnitus, hearing loss and MoCA values were statistically significant.*p < 0.05 indicates statistical significance. BMI, body mass index (kg/m^2^); MCI, mild cognitive impairment.

a Chi-squared test.

b Mann–Whitney U test.

### Univariate logistic regression screening for high risk factors

To verify the accuracy of risk factors, we applied univariate logistic regression to continue screening high risk factors for cognitive impairment. As shown in Table 3, the *p* values for age, hearing loss and tinnitus were statistically significant, and the OR values were both higher than 1. These results suggest that age, hearing loss and tinnitus are high risk factors for cognitive impairment. The results of our analysis suggest that older individuals with tinnitus have a 1.95 times higher risk of developing cognitive impairment compared with normal older people, while the risk of developing cognitive impairment increases by 1.08 times for every year of age. In contrast, in older patients with hearing loss, the risk of developing cognitive impairment increases by 1.09 times for each decibel of hearing loss.

**Table 3:** Univariate logistic regression analysis of the predictive effect of each variable on MCI.

Cognitive impairment	Odds ratio	Std. Err.	*z*	*p*	95% Conf.	Interval
Gender	0.96	0.23	−0.15	0.88	0.60	1.54
Age	1.08	0.02	3.6	0.00*	1.03	1.13
Height	0.98	0.16	−0.14	0.29	0.95	1.01
Body weight	0.98	0.01	−1.47	0.14	0.96	1.01
BMI index	0.96	0.03	−0.93	0.35	0.90	1.03
Hypertension	1.46	0.40	1.37	0.17	0.85	2.51
Diabetes	0.76	0.25	−0.81	0.41	0.40	1.45
Tinnitus	1.95	0.56	2.33	0.02*	1.11	3.43
Depression	1.27	0.43	0.71	0.48	0.65	2.48
Hearing loss	1.09	0.01	6.97	0.00*	1.06	1.12

Univariate logistic regression models found age, hearing loss and tinnitus to be high risk factors for mild cognitive impairment. Tinnitus (OR = 1.95) was a higher predictor than hearing loss (OR = 1.09).*Represents *p*-value less than 0.05.MCI, mild cognitive impairment; OR, odds ratio.

### Multi-factor logistic regression to develop a diagnostic model to evaluate the early diagnostic value of hearing loss and tinnitus on MCI

Univariate logistic regression screened variables including age, tinnitus and hearing loss were subjected to multifactorial logistic regression to ultimately screen for high-risk factors that may lead to cognitive impairment. Through multifactorial logistic regression analysis, we found that hearing loss and tinnitus were high risk factors for cognitive impairment as shown in Table 4. Our results identified hearing loss and tinnitus as potential risk factors for cognitive impairment, while age factors were excluded due to *p*-values greater than 0.05.

**Table 4:** Multi-factor logistic regression analysis of the predictive effect of each variable on MCI.

Cognitive impairment	Odds ratio	*p*	Cut off value	Sensitivity	Specificity	Correctly classified
Age	1.03	0.28				
Tinnitus	1.93	0.03*				
Hearing loss	1.09	0.00*	20	93.3%	34.7%	77.9%

Logistic regression models found hearing loss and tinnitus to be risk factors for mild cognitive impairment. Tinnitus (OR = 1.93) was a higher predictor than hearing loss (OR = 1.09).*represents *p*-value less than 0.05. MCI, mild cognitive impairment. Using a logistic regression algorithm, we calculated the Youden index of hearing loss, and the value of hearing loss corresponding to the maximum value of the Youden index was the cutoff value. Our results indicate that the risk of developing mild cognitive impairment increases by 9% when the hearing value is greater than 20 decibels.

**Figure 3:**
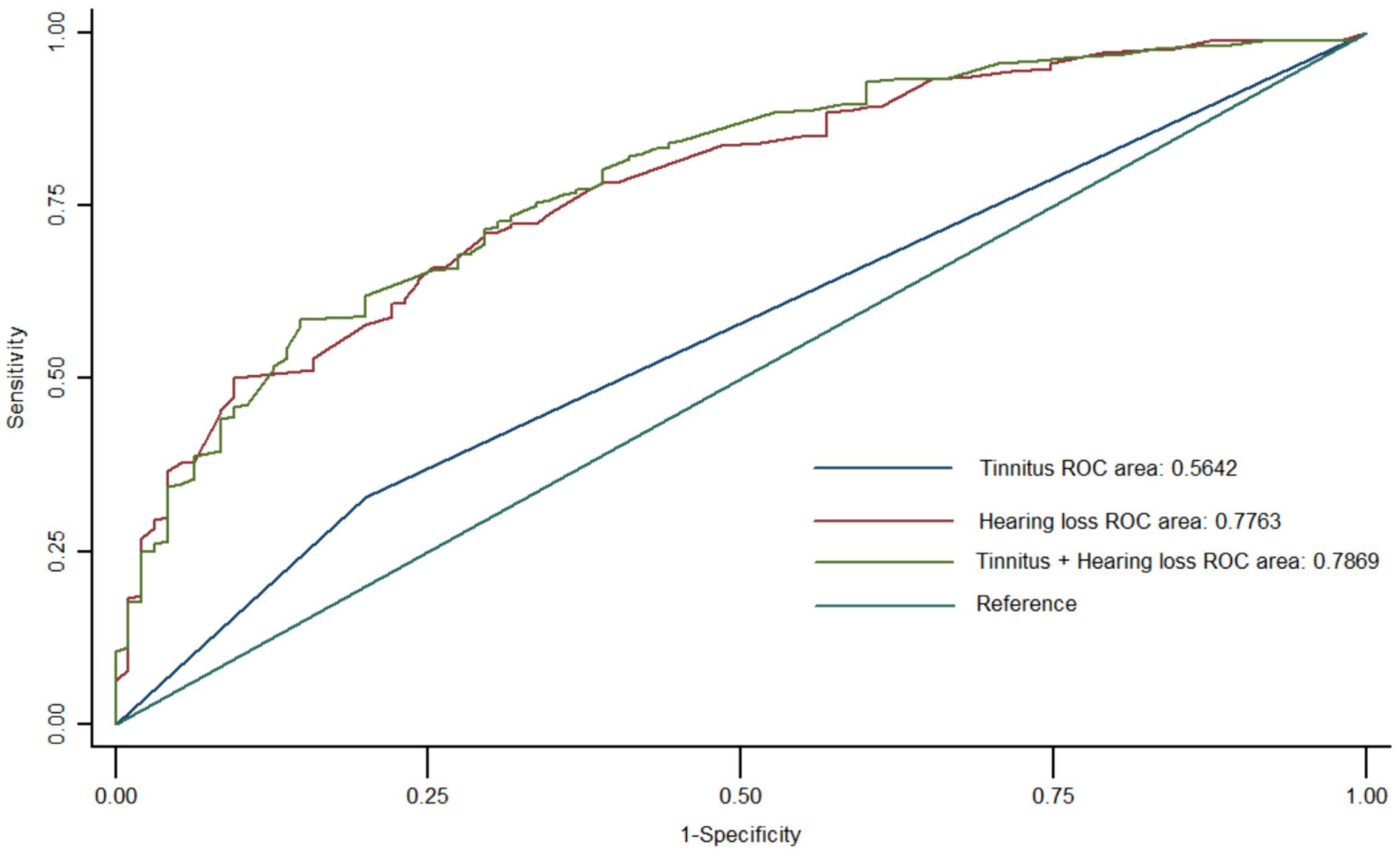
Multifactorial logistic regression model to evaluate the diagnostic efficacy of hearing loss and tinnitus on mild cognitive impairment. Hearing loss has a higher diagnostic efficacy than tinnitus for mild cognitive impairment. Hearing loss and tinnitus have the highest combined diagnostic effect on mild cognitive impairment.

In Figure 3 the ROC diagnostic model was constructed using logistic regression with cognitive impairment as the dependent variable and hearing loss and tinnitus as the independent variables. The results showed that the AUC for the diagnostic value of tinnitus on cognitive impairment was 0.56; the AUC for the diagnostic value of hearing loss on cognitive impairment was 0.77; and the AUC for the diagnostic value of the combined hearing loss and tinnitus on cognitive impairment was 0.78.

Using a logistic regression algorithm in Table 4, we calculated the Youden index of hearing loss, and the value of hearing loss corresponding to the maximum value of the Youden index was 20 decibels. Our results indicate that the risk of developing mild cognitive impairment increases by 9% when the hearing value is greater than 20 decibels.

### Multiple linear regression was used to explore the influencing factors of MCI

To verify the logistic regression results, multiple linear regression analysis was performed with MoCA as the dependent variable and pure-tone audiometry, HHIE-S, PHQ-9, age, and body mass index as independent variables (Table 5). The model results showed that the *p* value <0.001**, which proved that the model was successfully constructed. For variable collinearity performance, VIF was all less than 10, so the model did not have multiple collinearity problems and the model was well constructed. The results found that the independent variables PTA, HHIE-S and Age had an impact on the dependent variable MoCA. Combined with the B value, age had the greatest impact on MoCA.

**Table 5:** Results of multiple linear regression analysis of MoCA.

	Non standardized coefficient		Coefficient of standardization	*t*	*p*	VIF	*R* ^2^	Adjusted *R*^2^	*F*
	*B*	Standard error	Beta						
Constant	33.386	3.019	–	11.058	<0.001**	–	0.128	0.116	*F* = 10.511, *p* < 0.001**
PTA	−0.038	0.019	−0.112	−2.008	0.045*	1.273			
HHIE-S	−0.058	0.025	−0.127	−2.32	0.021*	1.226			
PHQ-9	0.058	0.069	0.043	0.842	0.400	1.066			
Age	−0.169	0.037	−0.243	−4.624	<0.001**	1.131			
BMI	0.071	0.067	0.053	1.056	0.292	1.021			

The results showed that the *p* value of the *F*-test was <0.001**, which proved that the model was successfully constructed. For variable collinearity performance, VIF was all less than 10, so the model did not have multiple collinearity problems and the model was well constructed. The results showed that the independent variables PTA, HHIE-S and age had an impact on the dependent variable MoCA. Combined with the B value, age had the greatest impact on MoCA. ** and * represent significance levels of 1 and 5%, respectively.PTA, pure tone audiometry; HHIE-S, hearing handicap inventory for the elderly-screening; PHQ-9, patient health questionnaire-9; BMI, body mass index.

## Discussion

Previous studies (Livingston et al., 2017; Chern and Golub, 2019; Livingston et al., 2020) have shown that MCI is a cause of AD, while both hearing loss and tinnitus are strongly associated with cognitive impairment. The cause of tinnitus is complex, may be emotional or fatigue, or tumor, or infection, or chronic diseases like diabetes or high blood pressure, or auditory nerve damage and so on. There are many confounding factors between tinnitus and hearing loss, as well as between tinnitus and cognitive function. Patients with tinnitus do not necessarily have hearing loss, while some patients with hearing loss are accompanied by tinnitus. Therefore, it is difficult to study the pathological relationship among tinnitus, hearing loss and cognitive impairment, and epidemiological investigation is one of the commonly used research methods. This study further validated the association of hearing loss and tinnitus with cognitive impairment through genetic and epidemiological studies across ethnic populations.

Mendelian Randomization is a data analysis technique for assessing etiological inferences in epidemiological studies that uses genetic variants with strong correlations with exposure factors as instrumental variables to assess causal relationships between exposure factors and outcomes (Emdin et al., 2017). In this study, the first Mendelian randomization method using SNPs as an instrumental variable was used to prove the causal relationship between HL and MCI, and it was found that the risk ratio of HL leading to MCI was 1.23. For the first time, we used Mendelian randomization to analyze the causal relationship between hearing loss and cognitive impairment. We identified 54 SNPs that were genetically strongly associated with hearing loss but not cognitive impairment, but whose expression changed when cognitive impairment developed. In the follow-up study, we can use these 54 SNPs as a combination to detect the patients with hearing loss by targeted sequencing, confirm and screen out the most significant SNPs to calculate the diagnostic cutoff value. If the patient’s SNPs value exceeds the cutoff value, it means that the risk of cognitive impairment is very high. At present, there is no biomarker for prediction and diagnosis of cognitive impairment in clinical practice. With the maturity of sequencing technology, the above SNPs may provide new ideas for early prevention and diagnosis of cognitive impairment.

On the basis of MR results, the correlation between HL and MCI were verified with the data from the Chinese checkups. This study initially explored an elderly population in the eastern coastal region of China. By analyzing factors such as pure tone hearing threshold, hearing loss, depression status, cognitive function scales, and chronic illness. Our study demonstrated a high prevalence of early cognitive impairment (73.8%) among Chinese older adults with suspected HL and tinnitus. In addition, this study confirmed the correlation between HL and tinnitus and early cognitive impairment, and explored the predictive and diagnostic ability of HL and tinnitus for early cognitive impairment. This study provides new ideas for the prevention of MCI.

A Brazilian census in 2023 evaluated data on 1,335 older adults which results found the prevalence of cognitive impairment was 20.5%. Older adults with hearing loss were 2.66 times more likely to have cognitive impairment than those without hearing loss (Paiva et al., 2023). A Japanese cross-sectional study using multivariate logistic regression analysis showed that HL was associated with MCI (odds ratio 1.60), independent of age, living alone, memory impairment, and impaired self-care (Saji et al., 2021). Correspondingly, our community survey was not a census, it was a specialized focus on a group of older adults suspected of having hearing loss or tinnitus, and our results demonstrate the higher prevalence of cognitive impairment in older adults suspected of having HL. Our results showed that an independent correlation between hearing loss and cognitive decline with an OR of 1.09. A recent meta-analysis of 34 studies reporting data on 48,017 participants found that people with hearing loss had an overall risk ratio of 1.44 for developing MCI (Mannarelli et al., 2017). Our findings also showed that older people with tinnitus had a 1.95-fold increased risk of MCI compared with normal older people, which is consistent with previous research findings (Brinkmann et al., 2021). In addition, our study did not find that factors such as weight and diabetes were associated with cognitive impairment, and the results are inconsistent with previous studies (Cox and Wallace, 2022). In this study, we calculated the diagnostic efficacy of hearing loss and tinnitus for early cognitive impairment by logistic regression and found that the diagnostic efficacy of hearing loss combined with tinnitus was consistent with the diagnostic efficacy of hearing loss alone for the diagnosis of disease. Subsequently, we calculated the cutoff value of hearing loss and found that the risk of developing mild cognitive impairment increased by 9% when the hearing value was greater than 20 decibels. While our study found that PTA, HHIE-S and age were the influencing factors of MoCA using multiple linear regression analysis, which was consistent with the results of our logistic regression.

The predictive analysis of MCI is complex, with social and clinical factors containing many interactions and covariates (Jang et al., 2017; Hu et al., 2021), and the analysis without proving causality is subject to many confounding factors. In this study, the genetic data of Europeans were first used to demonstrate the causal relationship between hearing loss as exposure factor and cognitive impairment as outcome through Mendelian randomization analysis (Yoneda et al., 2021). Subsequently, a cross-sectional study of the Chinese population was conducted to explore the risk factors for cognitive impairment. In this study, genome-wide association analysis combined with epidemiological investigation was used to explore and verify the risk factors of cognitive impairment in different ethnic groups, and the results have high reliability, providing a new idea for the prevention and early diagnosis of MCI patients.

However, this study has some limitations. First of all, the cognitive function assessment used in this study is an audio-based, oral oriented tool, and hearing loss may affect subjects’ performance during the cognitive assessment process, leading to an overestimation of cognitive decline in patients with hearing loss. Future in-depth research could be conducted by designing longitudinal cohort studies using cognitive function tools appropriate for patients with hearing loss. Second, results may be biased due to design flaws such as small sample sizes or different survey scale choices and different screening models. Third, hearing loss above 4000 Hz was not taken into account, which may cause some bias to the study results. Fourth, the etiology of tinnitus is very complex, and further clinical examination is needed to clarify the etiology of tinnitus. However, the tinnitus information in this study was obtained through the self-report of the respondents, and the results based on this analysis have limitations. In addition, future studies should provide a more specific description of tinnitus examinations in order to better understand the relationship between tinnitus and hearing loss and cognitive function. Finally, free community screening is not a census, which will lead to bias in the data. We hope that the results based on this study can attract the attention of the government, so as to conduct accurate research with the help of the government.

## Conclusion

Our Mendelian randomization and cross-sectional studies have showed that HL is a risk factor for MCI. The role of genetic, environmental, and lifestyle factors in the development and progression of hearing loss remains a major area of research in this field. More large-scale population-based epidemiologic surveys and clinical cohort studies should be conducted to explore early diagnosis and intervention strategies for HL based on lifelong hearing health, for reducing the incidence of cognitive impairment and improve the quality of life of the elderly population.

## Data availability statement

The original contributions presented in the study are included in the article/Supplementary material, further inquiries can be directed to the corresponding authors.

## Ethics statement

The studies involving humans were approved by the Ethics Committee of the Third People’s Hospital of Qingdao, affiliated with Qingdao University. The studies were conducted in accordance with the local legislation and institutional requirements. The participants provided their written informed consent to participate in this study.

## Author contributions

TX: Formal analysis, Investigation, Software, Writing – original draft. TZ: Investigation, Software, Writing – original draft. JL: Investigation, Methodology, Software, Writing – original draft. LZ: Investigation, Methodology, Supervision, Validation, Writing – review & editing. HG: Investigation, Methodology, Supervision, Writing – review & editing. RY: Conceptualization, Formal analysis, Investigation, Methodology, Supervision, Validation, Writing – original draft, Writing – review & editing. ZL: Conceptualization, Formal analysis, Funding acquisition, Investigation, Methodology, Software, Supervision, Validation, Writing – original draft.

## Footnotes

^1^https://gwas.mrcieu.ac.uk/
